# Echocardiographic Detection of Pulmonary Hypertension and Right Ventricular Failure in Infants with Bronchopulmonary Dysplasia: A Survey of the BPD Collaborative

**DOI:** 10.3390/children13050646

**Published:** 2026-05-05

**Authors:** Shilpa Vyas-Read, Shazia Bhombal, Roopa Siddaiah, Clifford L. Cua, Amulya Buddhavarapu, Robin L. McKinney, Philip T. Levy, Amanda L. Hauck, Nicolas F. M. Porta, Kathleen A. Gibbs, Krithika Lingappan, Matthew S. Douglass, Eric D. Austin, Steven H. Abman, Ronald W. Day

**Affiliations:** 1 Department of Pediatrics, Emory University, Children’s Healthcare of Atlanta, Atlanta, GA 30329, USA; 2 Department of Pediatrics, Arkansas Children’s Hospital, Little Rock, AR 72202, USA; 3 Department of Pediatrics, The Ohio State University, Nationwide Children’s Hospital, Columbus, OH 43205, USA; 4 Department of Pediatrics, The Warren Alpert Medical School of Brown University, Hasbro Children’s Hospital, Providence, RI 02903, USA; 5 Department of Pediatrics, Harvard Medical School, Boston Children’s Hospital, Boston, MA 02115, USA; 6 Department of Pediatrics, Northwestern University Feinberg School of Medicine, Lurie Children’s Hospital, Chicago, IL 60611, USA; 7 Department of Pediatrics, University of Pennsylvania Perelman School of Medicine, Children’s Hospital of Philadelphia, Philadelphia, PA 19104, USA; 8 Department of Pediatrics, University of Utah, Salt Lake City, UT 84112, USAronald.day@hsc.utah.edu (R.W.D.); 9 Department of Pediatrics, Vanderbilt University Medical Center, Nashville, TN 37232, USA; 10 Department of Pediatrics, University of Colorado, Children’s Hospital Colorado Anschutz Medical Campus, Aurora, CO 80045, USA

**Keywords:** bronchopulmonary dysplasia, echocardiography, pulmonary hypertension, right ventricular failure, screening for disease and disease severity

## Abstract

**Highlights:**

**What are the main findings?**

- Multi-disciplinary care teams who provide care for infants with BPD agree that echocardiography is feasible and reproducible for the diagnosis and treatment of BPD-PH in infants with bronchopulmonary dysplasia.
- Although most clinicians utilize clinical, laboratory and echocardiographic data in the diagnosis and treatment of infants with BPD at risk for PH, there is considerable variability in the parameters and cut-off thresholds that influence management.

**What are the implications of the main findings?**

- Improved team communications with shared understanding of test results, standardized echocardiographic protocols, and consistency in definitions may enhance the diagnosis and treatment of PH in infants with BPD.
- Risk-based models that incorporate the clinical, laboratory and echocardiographic data that are important to clinicians in the diagnosis and management of infants with BPD-PH should be developed.

**Abstract:**

Background: Echocardiography is a non-invasive test that is readily used to detect pulmonary hypertension associated with bronchopulmonary dysplasia (BPD-PH) and right ventricular failure (RVF). However, the most feasible, reproducible and accurate parameters to measure and use for guidance in addressing patient care have not been established and may differ between subspecialties. Methods: We surveyed members of the BPD Collaborative to determine how different care providers clinically evaluate infants for BPD-PH and RVF. Perceived challenges and obstacles that limit the utility of echocardiography are also reported. Results: Of the 108 survey respondents from ~45 centers, 55.6% were neonatologists, 18.5% were pediatric pulmonologists or pediatric intensive care physicians, 15.7% were pediatric cardiologists or pulmonary hypertension specialists, and 10.2% were other providers. Responses revealed discrepancies between specialists concerning the use of standard echocardiographic protocols and parameters that can be measured serially with relative ease, metrics that should be used to best define and distinguish the severity of BPD-PH or RVF, and parameter values that should be used to determine whether changes in PH-targeted medical therapies, hemodynamic or respiratory support are needed. Free text responses identified patient-, protocol-, cardiology-, technician-, and BPD-PH definition-related obstacles that may limit the reliable utility of echocardiography. Conclusions: Although most providers agree that echocardiography is feasible and of value, variability exists between subspecialists and centers, suggesting the need for improved standardization of imaging protocols and BPD-PH definition, consistent test interpretation, and effective communication of results to improve the reproducibility and accuracy of echocardiography in infants with BPD.

## 1. Introduction

Rates of bronchopulmonary dysplasia (BPD) in extremely premature newborns have remained ~40% despite significant research into its prevention and recent advances in neonatal care [1]. A literature review and meta-analysis in 2024 reported a pulmonary hypertension (PH) incidence of 5% in mild, 18% in moderate, and 41% in severe BPD [2]. The prevalence of PH associated with BPD (BPD-PH) may be as high as 60% in infants with severe BPD that require tracheostomy, and mortality for affected infants in the first 6 months of life approaches 40% [3,4]. The sequelae of BPD-PH and its complications persist beyond the neonatal period into adulthood. Limited data in survivors of prematurity suggest that abnormal pulmonary function, decreased exercise tolerance, disordered breathing and mild elevations in mean pulmonary artery pressure and total pulmonary resistance are present in over one-third of adult patients [5,6]. Although a proportion of premature infants have early vascular disease and are known to be at higher risk for BPD-PH during the neonatal period, approximately 17% may develop BPD-PH following hospital discharge [7,8]. Therefore, it is essential for neonatologists, pediatric pulmonologists, and pediatric cardiologists to develop algorithms to identify “at-risk” patients prior to neonatal intensive care (NICU) discharge to ensure adequate outpatient surveillance.

In response to these clinical concerns, the American Thoracic Society and the American Heart Association issued guidelines in 2015 recommending serial echocardiographic screening to detect and monitor PH in infants with BPD [9]. Although heart catheterization is used on an individualized basis for refractory cases of BPD-PH or to identify cardiovascular comorbidities, echocardiography is the mainstay for detecting and managing BPD-PH in the NICU as it is non-invasive and readily available. However, surveys of practicing neonatologists note significant discrepancies in the timing of echocardiography screening, the availability of standardized image acquisition, and the utilization of PH therapies in infants with BPD [10]. Common parameters used to assess pulmonary arterial pressures in the literature are the gradient of tricuspid valve regurgitation, configuration of the interventricular septum, pulmonary artery acceleration time (PAAT), left ventricular end-systolic eccentricity index (LVEIs), left ventricular maximum eccentricity index (LVEIm), and intracardiac shunt directionality/gradient [11,12,13,14,15,16]. Common echocardiographic parameters to assess right ventricular function in patients with BPD are the tricuspid annular plane systolic excursion (TAPSE), right ventricular fractional area change (RV-FAC) and right ventricular peak longitudinal strain (RV-LS) [17,18,19,20].

During meetings of the BPD-PH sub-group of the BPD Collaborative, a multi-disciplinary consortium of healthcare providers interested in the optimization of care for infants with BPD, we acknowledged the critical role of echocardiography in the evaluation and care of infants with BPD-PH. However, we noted a lack of standardization in image acquisition protocols, variability in provider interpretation, inconsistent threshold values used to indicate the presence or severity of BPD-PH, and poorly defined thresholds indicating a need for changes in therapy for infants with BPD [21]. Several reviews describe echocardiographic parameters that may be used to diagnose and monitor BPD-PH and right ventricular failure (RVF). However, the strengths and limitations of parameters are rarely noted and reported, a shortcoming that may impede progress in care and research. To assess the opinions of stakeholders and to determine if additional consensus is needed, we surveyed the members of the BPD Collaborative regarding their approach to the diagnosis of BPD-PH and RVF and the perceived obstacles that limit the feasibility, reproducibility, and accuracy of echocardiography in this patient population.

## 2. Materials and Methods

The BPD Collaborative is a multi-disciplinary consortium of physicians, advanced practitioners, nurses, respiratory therapists, nutritionists, pharmacists, fellows, and developmental and occupational therapists in pediatric sub-specialty fields that care for premature infants with BPD. At present, the BPD Collaborative consists of 60 centers, and 650 clinicians in the United States, Sweden, Japan, Canada, Italy, Holland, Germany, and Belgium. Members of the BPD-PH working group of the BPD Collaborative reviewed current literature and derived survey questions (Supplementary File S1). Single and multiple-choice questions were revised in monthly group meetings and again by email correspondence on two occasions. A test survey was sent to the BPD-PH sub-group in 2023 prior to uploading questions into the survey platform to ensure clarity with the question-and-answer choices. Institutional Review Board approval was through Emory University, and the survey was distributed from April to July 2024 to all members of the ~45 centers participating in the BPD Collaborative at that time. Recruitment for the survey occurred through monthly emails to the BPD Collaborative listserv.

The survey was administered and analyzed through the Qualtrics platform. Survey respondents were anonymous but were asked to self-identify their pediatric sub-specialty. Survey data regarding pediatric sub-specialties were categorized into 4 general provider categories: (1) neonatologists (NEO), (2) pediatric pulmonologists or pediatric intensive care specialists (PP/PICU), (3) pediatric cardiologists and pulmonary hypertension specialists (PC/PH), or (4) a multi-disciplinary group consisting of nurse practitioners, respiratory therapists, nurses, and “other” care providers (MULTI). Questions concerning the use of echocardiography in the assessment of BPD-PH were limited to patients without significant cardiovascular shunts. The responses for quantitative questions are shown as the percentage that chose a specific response over the number of responses in a specific specialty. To better understand potential barriers to echocardiography, respondents were asked to provide free text answers to 2 qualitative questions: (1) “What are the system-related, patient-related, and echocardiography-related obstacles to obtaining the measurements for pulmonary hypertension or right ventricular failure in infants with BPD?” and (2) “Please add any other comments regarding echocardiography for infants with BPD you’d like to communicate in the text box below.” Eighty-six free text comments were received to the 2 questions. Comments were initially grouped manually into predominant themes by SVR, and the major themes were ranked by the number of comments per topic/total number of comments. Grouping and implications of the major identified themes were discussed in meetings of the BPD-PH sub-group and shared with the manuscript authors for review and accuracy.

## 3. Results

### 3.1. Demographic Information for Respondents

Of the 431 individual members of the BPD Collaborative at that time, 108 (25.1%) responded to the survey. All responses for each survey question were captured, and not all respondents completed every question. The number of clinicians responding to each survey question is indicated in the top row of each Table, and denominators were variable due to non-response. The survey respondents were neonatologists (NEO) (n = 60, 55.6%), pediatric pulmonologists or pediatric intensivists (PP/PICU) (n = 20, 18.5%), pediatric cardiologists/pulmonary hypertension specialists (PC/PH) (n = 17, 15.7%), MULTI [n = 11, 10.2%, nurse practitioners (n = 4,3.7%), respiratory therapists (n = 1, 0.9%), nurses (n = 1, 0.9%), and “other” (n = 5, 4.6%)].

When asked which stakeholders should develop guidelines on the strengths and limitations of methods for diagnosing and monitoring BPD-PH, most respondents (92%) felt that pediatric pulmonary hypertension specialists would be ideal followed by neonatologists (87%). Ninety percent of respondents agreed that guidelines are needed to describe the most feasible, consistently reproducible, and accurate methods for identifying and monitoring PH and RVF in infants with BPD.

Ninety-two percent of respondents stated that their institution had a formal protocol or guidelines for screening and monitoring infants with BPD at risk for PH, and 86% of respondents reported that they can consult a PH team when neonatologists are concerned that an infant with BPD has PH. However, only 52% of respondents reported that the echocardiogram laboratory at their institution had a standard protocol for diagnosing and assessing BPD-PH.

### 3.2. Echocardiographic Parameters for PH and RVF in Infants with BPD

Respondents were asked to identify the clinical information providers use to determine whether an infant with BPD has PH or RVF (Table 1). All (100%) respondents use echocardiography to determine whether an infant has BPD-PH and RVF. Most respondents across all specialties also use historical information, physical findings, BNP or NT-proBNP values, and heart catheterization to evaluate for PH and RVF.

Table 2 lists the echocardiographic parameters that could be measured serially with reasonable ease and accuracy for the identification and monitoring of BPD-PH or RVF. Collectively, 63% of respondents reported the gradient of tricuspid valve regurgitation and 65% reported that a subjective assessment of septal flattening was sufficiently feasible, reproducible, and accurate to be used to identify and monitor PH. Most PC/PH respondents (71%) also reported that LVEIs would be useful. While only 58% of all respondents reported the BNP or NT-proBNP as useful in the longitudinal assessment of RVF, 100% of PC/PH respondents reported that these blood tests are sufficiently feasible, reproducible, and accurate. Sixty-nine percent of PC/PH respondents reported that the TAPSE is also a useful test for diagnosing and monitoring of RVF. The majority of NEO, PP/PICU and MULTI respondents reported variability in which tests would be appropriate in RVF management.

The survey identified considerable variability in responses regarding the cutoff values of systolic pulmonary arterial pressure that should be used to raise concern for the presence of PH in an infant with BPD across all provider categories (Table 3). Up to 29% of respondents reported that the ratio of the systolic pulmonary and systolic systemic arterial pressures should be used or were unsure of the appropriate cutoff.

Collectively, 83% of all respondents were unsure or reported that echocardiography cannot accurately grade BPD-PH severity (normal, mild PH, moderate PH or severe PH). However, 74% of all respondents reported that echocardiography can be used to distinguish between binary outcomes, such as normal versus abnormal pulmonary arterial pressures. The distribution of responses for identifying RVF was similar. However, a majority (57%) of PC/PH specialists reported that echocardiography can be used to distinguish between severity grades of RVF.

**Figure 1:**
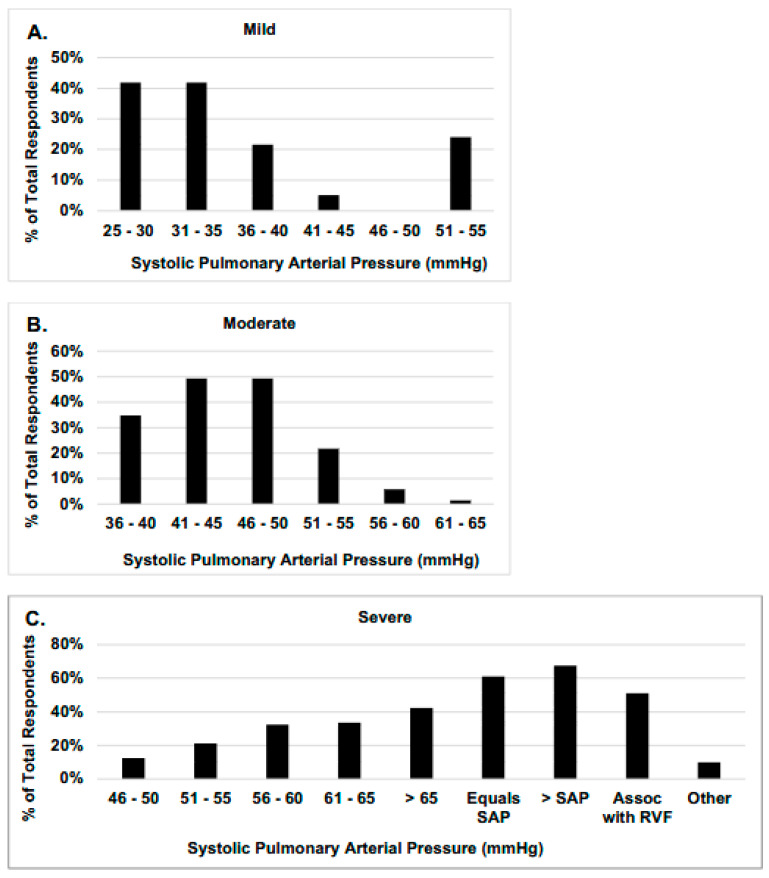
What range of estimated systolic pulmonary arterial pressure by echocardiography is consistent with mild, moderate, or severe pulmonary hypertension in an infant with BPD? Respondents provided a range of estimated systemic pulmonary arterial pressures consistent with mild (**A**), moderate (**B**) and severe (**C**) PH. moderate and severe PH. Excluding potential outliers, the overlap between reported values is not large with mild PH (25–40 mmHg), moderate PH (36–55 mmHg), and severe PH (>51 mmHg, ≥systemic arterial pressure, or any pressure associated with RVF). Percentages along the y—axis represent the number of times a range was chosen divided by the total responses for that question multiplied by 100. Assoc = associated; SAP = Systolic Systemic Arterial Pressure, RVF = Right Ventricular Failure.

Figure 1 shows the distribution of systolic pulmonary arterial pressures reported by respondents as consistent with mild, moderate, and severe PH. There was a range of potentially acceptable responses for each grade of severity with some overlap in the responses for mild PH (25–40 mmHg), moderate PH (36–55 mmHg), and severe PH (>51 mmHg, ≥systemic arterial pressure, or any pressure associated with RVF).

**Figure 2:**
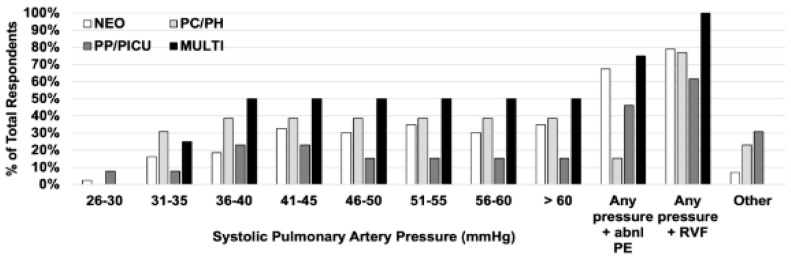
How high does a patient’s estimate of systolic pulmonary arterial pressure need to be to influence care? For example, at what level would you consider starting a medication to treat pulmonary hypertension or adjust respiratory support?

Table 4 lists the echocardiographic estimates of PH severity that respondents stated were needed to guide clinical decisions about the care of infants with BPD. More than two-thirds of respondents in all categories agreed that the systolic pulmonary arterial pressure and the ratio of systolic pulmonary to systolic systemic arterial pressures are needed. Figure 2 illustrates considerable variability in the estimates of systolic pulmonary arterial pressure that respondents would use to influence care. Less than 50% of respondents indicated that interventions, including the potential initiation of medical therapy or changes in respiratory support, should be considered when PH is only mildly or moderately severe.

Respondents provided a range of estimated systemic pulmonary arterial pressures that felt would influence decisions to modify the care of the infants with BPD. Approximately 1/3 of neonatologists (NEO) or pediatric cardiologists/pediatric pulmonary hypertension specialists (PC/PH) agreed that interventions including the initiation of medical therapy or changes in respiratory support should be considered when systolic pulmonary arterial pressure is >40 mmHg, and a majority of respondents in all specialties agreed that any pressure with symptoms of RVF should be treated. Percentages along the y-axis represent the number of times a range was chosen divided by the total responses for that question multiplied by 100. PP/PICU = pediatric pulmonology and pediatric intensive care specialists; MULTI = Advanced practitioners, nurses, respiratory therapists; Abnl PE = Abnormal Physical Examination, RVF = Right Ventricular Failure.

### 3.3. Qualitative Themes Regarding Obstacles in Echocardiography

Table 5 lists the major themes in response to the question, “What are the system-related, patient-related, and echocardiography-related obstacles to obtaining the measurements for pulmonary hypertension or right ventricular failure in infants with BPD?” Qualitative responses highlighted several factors that may affect the feasibility, reproducibility and accuracy of echocardiographic measurements. These responses were collated and analyzed qualitatively. After review, comments were grouped into 5 major themes that emerged from the data: Patient, Echo Protocol, Cardiologist, Technician, and PH Definition. Patient-related themes suggest that measurement reliability may be limited by patient agitation or the severity of lung disease that obscures cardiac windows, even when bedside imaging is technically feasible. Protocol-related themes highlight the lack of standardized imaging protocols or consensus definitions, contributing to variability in measurements. Cardiologist-related themes include differences in expertise and interpretation, as well as opportunities to develop a collaborative relationship and to communicate a shared understanding of test results among multidisciplinary providers. The technician-related themes highlight concerns about the availability of trained personnel and variability in technical skill or familiarity with imaging protocols specific to BPD-PH or RV dysfunction. Finally, respondents emphasized that the absence of a universally accepted definition of BPD-PH limits the interpretability and consistency of echocardiographic assessments and complicates efforts to align multidisciplinary groups.

## 4. Discussion

This survey was performed by the BPD Collaborative to assess the role of echocardiography in the diagnosis and management of BPD-PH in premature infants. Echocardiography is routinely employed to assess PH and RVF by all respondents who care for patients with BPD. However, uniform protocols for screening and reporting BPD-PH and RVF are not available in all institutions. Additionally, there is inconsistency in the echocardiographic parameters, the definitions of PH, and the parameters and thresholds that are used to influence the management of BPD patients by providers. Qualitative responses also indicated that several obstacles limit the reliability of echocardiography and the multi-disciplinary team’s shared understanding of patient management. Ninety percent of respondents agreed that guidelines should be refined to better identify and monitor BPD-PH and RVF.

### 4.1. Feasibility, Reproducibility and Accuracy of Echocardiographic Parameters for PH and RVF

Collectively, 63% of respondents reported that the gradient of tricuspid valve regurgitation is an appropriate parameter to measure. However, many patients with BPD do not have enough tricuspid valve regurgitation to accurately estimate the systolic pulmonary arterial pressure and it is often not reliably measured in agitated infants [22]. Similarly, many patients do not have enough pulmonary valve insufficiency to estimate mean and diastolic pulmonary arterial pressures. Thus, pressure gradients are not reproducible or accurate in this patient population.

Approximately two-thirds of respondents (65%) reported that a subjective assessment of septal flattening is an appropriate parameter to measure. Septal flattening has been shown to be associated with BPD-PH that leads to worse outcomes and mortality for infants with BPD [23]. However, septal flattening is prone to subjective interpretation, especially when utilized to diagnose pulmonary vascular disease, and accuracy is limited if post-tricuspid shunts are present [24]. The LVEIs or LVEIm may provide more quantitative assessments of right ventricular systolic pressure [11,14,15]. The LVEIs and LVEIm are not difficult to measure; however, these parameters have some shortcomings. Inaccuracy may occur if perpendicular dimensions of the left ventricle are measured from the irregular, inner endocardial surface instead of the less deformable inner muscular margin of the left ventricle. Off-axis views of the ventricle may also limit reproducibility and accuracy. Most studies have reported values of LVEIs or LVEIm that correlate with percentages of the systolic systemic arterial pressure. Unfortunately, a given percentage of systolic systemic arterial pressure may correspond with markedly different systolic pulmonary arterial pressures because the systolic systemic arterial pressure is not a constant variable for individual or different patients (e.g., half systemic pulmonary arterial pressure is 50 mmHg if the systolic systemic arterial pressure is 100 mmHg and 35 mmHg if the systolic systemic arterial pressure is 70 mmHg). The systolic systemic arterial pressure must be measured at the time of echocardiography and then used to derive the estimated systolic pulmonary arterial pressure for the LVEIs or LVEIm to be more accurate and clinically useful. Another potential shortcoming of the LVEIs is a decrease in correlation between measurements and pressures when pulmonary arterial pressures are high [14]. Prospective studies of this patient population would allow for better determination of the values of LVEIs or LVEIm that are most appropriate to guide clinical care.

PAAT and indexed PAAT may be used to identify and monitor BPD-PH and associated outcomes [17,25,26,27]. In our survey, only 19% to 26% of respondents reported that the PAAT or indexed PAAT alone are appropriate parameters to measure. The PAAT and indexed PAAT are not difficult to measure. However, PAAT values correlate better with values of systolic pulmonary arterial pressure and pulmonary vascular resistance when the pulmonary arterial pressure is low than when it is elevated. Further, PAAT has a low sensitivity for diagnosing elevated pulmonary vascular resistance [28]. More research is needed to determine the values of PAAT or indexed PAAT that should be used to guide clinical care.

Many subspecialists reported uncertainty concerning the reliability of tests for RVF. However, 100% and 69% of PC/PH respondents reported that values of BNP or NT-proBNP and TAPSE are appropriate tests to evaluate for the presence of RVF, respectively. The BNP or NT-proBNP are more reliable tests to measure RVF that accompanies PH, rather than PH alone [29,30]. A multi-modal imaging approach with echocardiography and magnetic resonance imaging may improve the accuracy of monitoring RVF; however, magnetic resonance imaging is often not feasible in infants with BPD [31]. More research is needed to determine whether subjective assessments of RVF, TAPSE, RV-FAC, or RV-LS are more feasible, reproducible or accurate than values of BNP or NT-proBNP.

As guidelines for the use of echocardiography to detect and monitor PH and RVF in infants with BPD evolve, providers should carefully consider the strengths and limitations of each parameter. Although echocardiography is more available longitudinally, it may not be the most reliable or accurate test for all patients and other hemodynamic or imaging modalities may need to be considered to detect or manage PH with or without RVF in infants with BPD.

### 4.2. Distinguishing Between Degrees of PH and RVF Severity

Most respondents felt that systolic pulmonary arterial pressure between 25 and 35 mmHg, 40–50 mmHg, and >65 mmHg or ≥systolic systemic arterial pressure were appropriate ranges to define mild, moderate and severe PH, but there was considerable overlap between selected ranges. However, only ~40% of pediatric cardiologists and pulmonary hypertension specialists responded that systolic pulmonary arterial pressures > 40 mmHg would influence their care of the infant with BPD, highlighting an uncertainty around the optimal cut-off to change therapy, and the possible presence of variables other than pressure measurements in their decision-making. In support of this, the majority of pediatric cardiologists and pulmonary hypertension specialists were only able to clearly indicate a need for change in therapy when the systolic pulmonary arterial pressure was combined with signs of RVF. These findings demonstrate the idea that although systolic pulmonary arterial pressure measurements are important in guiding clinical care, they are unlikely the sole factor driving therapeutic change.

While echocardiography may not be able to sharply distinguish all grades of PH severity, many respondents reported that it may be able to distinguish between the absence and presence of PH, and the absence or presence of RVF. This ability may be particularly useful if interventions to treat PH or RVF are beneficial, even with early or mild disease. Additionally, echocardiography is unable to reliably distinguish precapillary PH from postcapillary or combined PH, and cardiac catheterization may be necessary in some cases. When invasive hemodynamic measurements are performed, a mean pulmonary arterial pressure, a pulmonary arterial wedge pressure or left atrial pressure, and a calculation of pulmonary blood flow are used to define PH. Future research should focus on determining the universally accepted definitions for the grade of PH and RVF severity that is needed to guide decisions in clinical care and indications for further non-invasive or invasive imaging modalities.

### 4.3. Obstacles Limiting the Feasibility, Reproducibility and Accuracy of Echocardiography

Free text responses highlighted several factors that may affect the feasibility, reproducibility, and accuracy of echocardiographic measurements. Some Patients-related concerns may be difficult to resolve due to imaging problems associated with lung disease, sedation, or the lack of sedation. Protocol-, Cardiologist- and Technician-related concerns may be resolved by improved standardization of imaging protocols, improved expertise, consistent interpretation, and improved communication to allow for a shared understanding in test results. Finally, multi-disciplinary groups need universally accepted definitions of PH and RVF in infants with BPD.

### 4.4. Opportunities to Eliminate Gaps in Knowledge and Enhance the Utility of Echocardiography for Evaluation of PH and RV in Infants with BPD

**Figure 3:**
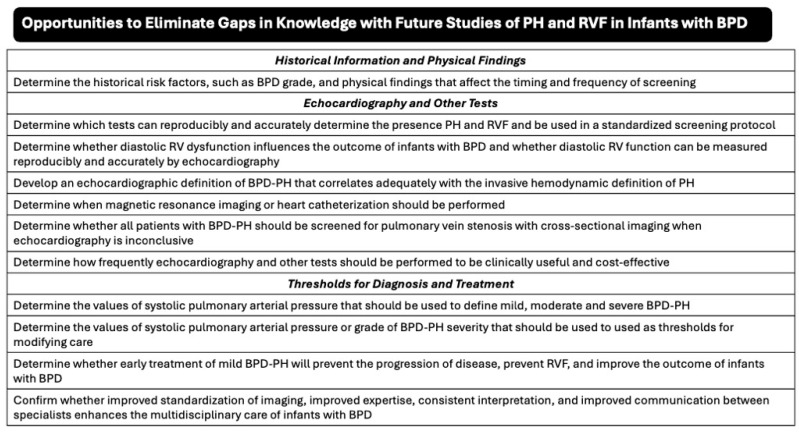
Gaps in knowledge for screening of infants with BPD for pulmonary hypertension (PH) and right ventricular (RVF). Survey findings highlighted opportunities in clinical factors, echocardiographic parameters, and thresholds for diagnosis and treatment that should be evaluated to determine optimal screening for infants with BPD.

Survey respondents showed considerable variability in their responses and may not have responded correctly to individual questions. While respondents agreed that certain clinical, laboratory and echocardiographic data are important to obtain serially in infants with BPD, these areas of agreement do not provide enough evidence to propose a framework for best practices or guiding principles. Care providers still need to refine guidelines for testing, agree upon the diagnoses of PH and RVF, and determine when medical therapy or other interventions should be used in infants with BPD. At this time, we can only list some of the gaps in knowledge that need to be resolved in future studies for multidisciplinary teams to ultimately provide better care (Figure 3). Until our judgment improves, care providers may need to acknowledge the limitations of individual tests and cautiously integrate the results of several tests to make appropriate clinical decisions.

### 4.5. Survey Limitations

We are aware that PH is a multifactorial disease in patients with BPD. A comprehensive discussion concerning the ability of echocardiography to assess and distinguish the relative roles of lung disease, precapillary vascular disease, shunts with high flow, and postcapillary disease is beyond the scope of this report. Here, we describe some of the limitations in the survey and the interpretation of the results.

- We recognize that the generalizability of the survey findings is limited by a potential ascertainment bias that is introduced by the low but acceptable response rate. Identification of respondents by email or center was not collected to protect respondent privacy. Additionally, the institutions that participate in the BPD Collaborative are largely academic, university centers, and we did not survey sub-specialists in private practice settings. We identified a genuine problem in practice variation with the use and interpretation of echocardiographic parameters for BPD-PH and RVF in our cohort. Respondents reported a need for standardization in imaging protocols; however, our findings only confirm a need for future efforts to improve the reproducibility and accuracy of current tests, or to develop new tests.
- Respondents were not asked if they believe a combination of tests, such as BNP or NT-proBNP in addition to echocardiography, might detect and monitor BPD-PH or RVF more effectively than echocardiography alone. Indeed, many respondents reported the use of observations and tests other than echocardiography. The use of clinical findings, electrocardiography, CT angiography, magnetic resonance imaging, and heart catheterization may complement incomplete information from echocardiography.
- We cannot fully address whether standardization of protocols and improved imaging will surely improve the accuracy of echocardiography without a comparison to heart catheterization. Less than half of infants with BPD undergo heart catheterization and invasive hemodynamic measurements to assess anatomy, vasoreactivity, and shunts [32]. While invasive hemodynamic measurements are typically performed to confirm the diagnosis and severity of PH in other PH populations, heart catheterization may not be an ideal standard for the assessment of PH in patients with BPD. Direct pressure measurements can be performed; however, estimates of pulmonary blood flow by thermodilution and the Fick principle may not be accurate with persistent shunts or discrepancies in pulmonary venous oxygenation, respectively.
- We did not explore whether respondents rely upon indices derived from more than one echocardiographic parameter. The ratio of TAPSE and estimated right ventricular systolic pressure provides an assessment of ventricular and vascular interactions, which correlates with death in infants with congenital diaphragmatic hernia [33]. Pressure and strain measurements can be integrated to describe global right ventricular myocardial work [34]. However, there was enough variability and uncertainty in our survey with single parameters to raise concern that indices derived from multiple parameters may not be sufficiently feasible, reproducible or accurate in infants with BPD.
- We did not ask whether the severity of BPD impacts the consideration of echocardiographic parameters of BPD-PH and RVF in infants with BPD. Given the importance of cardiopulmonary interactions, it is conceivable that parameters of PH and RVF could be interpreted differently by different subspecialists in infants with more severe airspace and/or airway disease who receive higher ventilatory pressures.
- While previous studies have examined the optimal timing of tests to identify and monitor BPD-PH and RVF, we did not ask respondents to comment on when or how often screening should be performed [35,36]. Many institutions are screening at a postmenstrual age of 36 weeks. However, BPD-PH may develop after an initial evaluation. Further, pulmonary vein stenosis may occur after initial screening and may be overlooked if not thoroughly evaluated by echocardiography and cross-sectional imaging after a gestational age of 36 weeks. In clinical practice, longitudinal assessment can be an important factor leading to diagnosis and subsequent management of these patients, especially if the reliability of the findings on any given study is in question. Prospective studies are needed to examine the ideal timing of screening echocardiograms to accurately identify pulmonary hypertension and improve patient outcomes.
- Questions concerning the cost effectiveness of testing modalities were not included in our survey. Echocardiography is a moderately expensive test, though less expensive than magnetic resonance imaging and heart catheterization. More research is needed to determine the appropriate frequency to identify problems and monitor response to interventions without excessively increasing cost.

## 5. Conclusions

Most providers who are members of a multi-disciplinary program caring for infants with severe BPD agree that echocardiography is the most feasible method to identify and manage BPD-PH. The reproducibility and accuracy of echocardiography may be improved with an achieved consensus regarding test limitations, standardization of imaging protocols, consistent test interpretation, and effective communication of results between care providers. While most multi-disciplinary teams utilize a composite of clinical, laboratory and echocardiographic studies to determine the diagnosis and treatment thresholds for the care of infants with BPD, the optimal parameters and cut-off thresholds to diagnose and treat BPD-PH in premature infants are still unclear and warrant further investigation.

## Figures and Tables

**Table 1:** What clinical information and tests do you use to determine whether a patient with BPD has pulmonary hypertension or right ventricular failure?

**Pulmonary Hypertension**	**TOTAL**	**NEO**	**PC/PH**	**PP/PICU**	**MULTI**
**Total (n)**	**94**	**55**	**14**	**17**	**8**
Historical information	67%	66%	71%	71%	63%
Physical examination	64%	64%	79%	53%	63%
Blood tests (BNP or NT-proBNP)	68%	71%	57%	65%	75%
Electrocardiogram	19%	15%	36%	18%	25%
Chest x-ray	21%	16%	21%	24%	50%
Echocardiography	100%	100%	100%	100%	100%
Heart catheterization	67%	60%	79%	88%	50%
**Right Ventricular Failure**	**TOTAL**	**NEO**	**PC/PH**	**PP/PICU**	**MULTI**
**Total (n)**	**93**	**54**	**14**	**17**	**8**
Historical information	60%	59%	71%	53%	63%
Physical examination	62%	65%	86%	47%	38%
Blood tests (BNP or NT-proBNP)	63%	67%	79%	53%	38%
Chest X-ray	29%	32%	14%	29%	38%
Echocardiography	100%	100%	100%	100%	100%
Heart catheterization	52%	50%	36%	65%	63%

Respondents could choose as many options as were applicable to this question regarding the information or tests that would help them determine if a patient had pulmonary hypertension or right ventricular failure. The left-hand column demonstrates the options that were available. Respondents were categorized into 4 groups by specialty: NEO = neonatologist; PC/PH = pediatric cardiology, pulmonary hypertension specialist; PP/PICU = pediatric pulmonology, pediatric intensive care; MULTI = a combination of nurses, respiratory therapists, advanced practice providers, and others. Results for “magnetic resonance imaging” and “other” were selected by ≤25% of respondents and are not shown. Percentages reflect the number of times a response was chosen over the total responses in that category multiplied by 100. BNP = B-type natriuretic peptide.

**Table 2:** In infants with BPD, which of the following parameters can be measured serially with reasonable ease and accuracy, allowing them to be useful in the identification and monitoring of PH or right ventricular failure?

**Echocardiographic Parameters for PH**	**TOTAL**	**NEO**	**PC/PH**	**PP/PICU**	**MULTI**
**Total Count (n) **	**91**	**53**	**14**	**17**	**7**
Gradient of tricuspid valve regurgitation	63%	64%	64%	65%	43%
Peak gradient of pulmonary valve insufficiency	34%	34%	50%	24%	29%
End-diastolic gradient pulmonary valve insufficiency	22%	15%	50%	24%	14%
A subjective assessment of septal flattening	65%	59%	86%	65%	71%
Pulmonary artery acceleration time	26%	28%	36%	18%	14%
Pulmonary artery acceleration time, indexed for heart rate or ejection time	19%	19%	21%	12%	29%
End-systolic left ventricular eccentricity index	35%	30%	71%	29%	14%
Maximum left ventricular eccentricity index	25%	25%	43%	24%	0%
Right ventricular anterior or inferior wall thickness	37%	38%	36%	41%	29%
Not sure	36%	38%	0%	47%	71%
**Parameters for RVF**	**TOTAL**	**NEO**	**PC/PH**	**PP/PICU**	**MULTI**
Total Count (n)	90	53	13	17	7
Tricuspid Annular Plane Systolic Displacement	43%	40%	69%	41%	29%
Right Ventricular Fractional Area Change	30%	28%	54%	18%	29%
Tissue Doppler imaging	14%	15%	23%	12%	0%
Right Ventricular Strain	29%	32%	31%	24%	14%
BNP or NT-proBNP	58%	49%	100%	59%	43%
Not sure	49%	55%	8%	53%	71%

Respondents could choose as many options as were applicable to this question. The left-hand column demonstrates the options that were available. Respondents were categorized into 4 groups by specialty: NEO = neonatologist; PC/PH = pediatric cardiology, pulmonary hypertension specialist; PP/PICU = pediatric pulmonology, pediatric intensive care; MULTI = a combination of nurses, respiratory therapists, advanced practice providers, and others. Responses for “Other” categories for parameters for BPD-PH and RVF were selected <20% of the time. Percentages reflect the number of times a response was chosen over the total responses in that category multiplied by 100. PH = pulmonary hypertension; RVF = right ventricular failure; BNP = B-type natriuretic peptide.

**Table 3:** What estimated systolic pulmonary arterial pressure by echocardiography should be used as a cutoff to raise concern for the presence of pulmonary hypertension in an infant with BPD?

**Systolic Pulmonary Arterial Pressure Cut-off (mmHg)**	**TOTAL**	**NEO**	**PC/PH**	**PP/PICU**	**MULTI**
Total Count	86	50	14	16	6
25	19%	10%	21%	44%	17%
26–30	23%	24%	14%	25%	33%
31–35	26%	30%	21%	13%	33%
36–40	7%	8%	14%	0%	0%
>40	6%	8%	0%	0%	17%
Other—Free Text Responses	20%	20%	29%	19%	0%

Respondents could choose one option to this question. The left-hand column demonstrates the options that were available. Free text responses included: “>1/2 systemic level” or “it should be based on percent of systemic bp,” “+Right Atrial Pressure (~5 mmHg),” “>1/3 systemic systolic BP,” and “Not sure.” Respondents were categorized into 4 groups by specialty: NEO = neonatologist; PC/PH = pediatric cardiology, pulmonary hypertension specialist; PP/PICU = pediatric pulmonology, pediatric intensive care; MULTI = a combination of nurses, respiratory therapists, advanced practice providers, and others. Percentages reflect the number of times a response was chosen over the total responses in that category multiplied by 100. Free text responses are listed at the bottom of the left-hand column. BP = systolic blood pressure.

**Table 4:** Which echocardiographic estimates of the severity of pulmonary hypertension need to be reported in order to guide clinical decisions about the care of infants with BPD?

**Echocardiographic Parameters**	**NEO**	**PC/PH**	**PP/PICU**	**MULTI**
Total Count (n)	46	13	15	4
Systolic pulmonary arterial pressure	70%	85%	67%	75%
Mean pulmonary arterial pressure	52%	39%	60%	75%
Diastolic pulmonary arterial pressure	24%	15%	13%	75%
Ratio of systolic pulmonary arterial pressure to systolic systemic arterial pressure	80%	77%	73%	75%
Pulmonary vascular resistance	48%	0%	33%	50%

Respondents could choose as many options as were applicable to this question. The left-hand column demonstrates the options that were available. Respondents were categorized into 4 groups by specialty: NEO = neonatologist; PC/PH = pediatric cardiology, pulmonary hypertension specialist; PP/PICU = pediatric pulmonology, pediatric intensive care; MULTI = a combination of nurses, respiratory therapists, advanced practice providers, and others. Respondents answered, “subjective categorization of severity,” and “other” ≤ 25% of the time. Percentages reflect the number of times a response was chosen over the total responses in that category multiplied by 100.

**Table 5:** Major Themes in response to the question: “What are the system-related, patient-related, and echocardiography-related obstacles to obtaining the measurements for pulmonary hypertension or right ventricular failure in infants with BPD?”.

Patient N = 32 (37%)	“Poor acoustic windows, uncooperative patients”; “Sedated versus non-sedated consistency in reporting consistency of objective findings”; “Infants wakefulness at the time; or agitation”; “Poor windows due to pulmonary overexpansion/air trapping”; “Not obtaining the echo at the infant’s baseline status, echos obtained during an exacerbation of BPD tend to show increased severity of PH”; “Severity of lung disease (resulting in difficulty in acquiring optimal echocardiographic data)”; Respiratory support (impact of adequate vs. inadequate respiratory support on PH)”
Echo Protocol N = 30 (35%)	“Lack of standardized echo protocols”; “Absence of TR and reliance on subjective impression of septal flattening”; “Lack of consensus on when and how frequently to monitor ECHOs”; “Lack of consistency in echocardiography measurements”; “Lack of consistent echocardiographic findings reported and measured relevant to assessment of pulmonary hypertension”; “Unless your team has a standard approach to assess and review TTEs, variability within an ECHO imaging room may result in variability in interpretations”
Cardiologist N = 21 (24%)	“Inconsistent echocardiography reports by different cardiologists”; “Expertise among interpreting cardiologists”; “Need to improve communication between cardiology and neonatology so that we have a shared understanding condition…”; “Multiple different echo reading attendings per study”; Measurements not performed every study, or not reported in final read”; “Inconsistency in echo acquisition and interpretation”; “Lack of knowledge of different PH indices by the echo reader”
Technician N = 19 (22%)	“Lack of training on standardized measurements”; “Inadequate data reported by sonographers”; “Lack of consistent image acquisition by sonographer (i.e., no pulmonary artery spectral Doppler, no TAPSE or TDI, off-axis parasternal views)”; “Variability in staffing and technique for sonographers”; “Consistency of technique at the bedside”; “Availability of pediatric echo trained sonographers in outside institutions”
PH Definition N = 12 (14%)	“Add indications when to start screening, and how frequent should echos be done for surveillance”; “Agreement between stakeholders”; “Definitions for PH (including severity, timing, longitudinal trajectory, resolution)”; “Indications for initiation and cessation of therapies/treatments (including definitions of “success” and “failure”)”; “Discrepancies amongst expert recommendations; not applying the phenotypes of chronic PH to the management of patients; limitation in knowledge of newer echocardiographic markers for PH assessment”; “Subjectivity in mild vs moderate vs severe PH, various other factors affecting measurements and degree of PH at any point in time”; “Lack of standardized approach or diagnosis and management of BPD-PH”

The table illustrates the 5 major themes that arose from 86 responses to this question. Representative quotations are listed to the right of the theme, which is indicated in the left-hand column. “Other” comments that did not fit into a dominant theme included concerns around lack of access to echocardiography or heart catheterization, other clinical factors, neonatal co-morbidities influencing diagnosis, the dynamic nature of the echocardiogram, difficulty visualizing pulmonary veins, and cost. BPD = bronchopulmonary dysplasia; Echo = echocardiogram; PH = pulmonary hypertension; TR = gradient of tricuspid regurgitation; TTE = transthoracic echocardiography; TAPSE = Tricuspid Annular Plane Systolic Displacement; TDI = Tissue Doppler Imaging.

## Data Availability

Data available on request due to restrictions in participant privacy mandated by the Institutional Review Board at Emory University. The data presented in this study are available on request from the corresponding author.

## Supplementary Materials

The following supporting information can be downloaded at: https://www.mdpi.com/article/10.3390/children13050646/s1, File S1: Survey.
