# Supplementary material for: Echocardiographic Detection of Pulmonary Hypertension and Right Ventricular Failure in Infants with Bronchopulmonary Dysplasia: A Survey of the BPD Collaborative
Source: Children (Basel). 2026 May 5;13(5):646. doi: 10.3390/children13050646 (PMC13204351; doi:10.3390/children13050646)
Supplement: Supplementary file 1 [file children-13-00646-s001.zip › children-4269598-supplementary.pdf]

EMORY UNIVERSITY

Block 1

FOCUS 1: Who are the best stakeholders for a white (position) paper describing methods to diagnose, monitor and consider treatment of pulmonary hypertension and right ventricular failure in infants with bronchopulmonary dysplasia (BPD)?

1. What is your role in the care of infants with BPD? Check all that apply.

- ○ Neonatologist
- ○ Pulmonary or sleep medicine specialist
- ○ Pediatric cardiologist
- ○ Pediatric pulmonary hypertension specialist
- ○ Pediatric intensive care specialist
- ○ Nurse practitioner
- ○ Nurse
- ○ Pharmacist
- ○ Respiratory therapist
- ○ Sonographer
- ○ [ ] Other

2. Who are the best stakeholders for developing a white paper or position paper to describe the strengths and limitations of methods to diagnose and monitor pulmonary hypertension and right ventricular failure in infants with BPD? Check all that apply.

- ☐ Neonatologists
- ☐ Pulmonary or sleep medicine specialist
- ☐ Pediatric cardiologists (all)
- ☐ Pediatric cardiologists (echocardiographers only)

- ☐ Pediatric pulmonary hypertension specialists
- ☐ Pediatric intensive care specialist
- ☐ Nurse practitioners
- ☐ Pharmacists
- ☐ Respiratory therapists
- ☐ Nurses
- ☐ Sonographers
- ☐ Other

3. For infants who do **not** have a history of pulmonary hypertension and are **not** on vasodilator therapy, does your institution have a formal protocol or guidelines for screening and monitoring infants with established BPD for pulmonary hypertension?

- ◯ Yes
- ◯ No
- ◯ Not sure

4. Does your institution have a pulmonary hypertension team that can be consulted when neonatology providers are concerned that an infant with BPD has pulmonary hypertension?

- ◯ Yes
- ◯ No
- ◯ Not sure

Block 2

FOCUS 2: Evaluation of Pulmonary Hypertension and Right Ventricular Function in Infants with BPD

5. What clinical information and tests do you use to determine whether a patient with BPD has **pulmonary hypertension**? Check all that apply.

- ☐ Historical information (poor feeding, low oxygen saturation measurements, poor growth, etc.)
- ☐ Physical examination (respiratory effort, heart sounds, perfusion, etc.)
- ☐ Blood tests (BNP or NT-proBNP)

- ☐ Electrocardiogram
- ☐ Chest x-ray
- ☐ Echocardiography
- ☐ Magnetic resonance imaging
- ☐ Heart catheterization
- ☐ [ ] Other

6. What clinical information and tests do you use to determine whether a patient with BPD has **right ventricular failure**? Check all that apply.

- ☐ Historical information (poor feeding, low oxygen saturation measurements, poor growth, etc.)
- ☐ Physical examination (respiratory effort, heart sounds, perfusion, etc.)
- ☐ Blood tests (BNP or NT-proBNP)
- ☐ Electrocardiogram
- ☐ Chest x-ray
- ☐ Echocardiography
- ☐ Magnetic resonance imaging
- ☐ Heart catheterization
- ☐ [ ] Other

7. Does the Echo Lab at your institution have a standard protocol for diagnosing and assessing the severity of pulmonary hypertension in patients with BPD?

- ○ Yes
- ○ No
- ○ Not sure

8. Would the Echo Lab at your institution be amenable to having a standard protocol for using echocardiography to assess pulmonary hypertension consistently in infants with BPD?

- ○ Yes
- ○ No
- ○ Not sure

9. Echocardiography is the most **feasible** method to identify and serially monitor *pulmonary hypertension* in infants with BPD at the bedside.

- Agree
- Disagree
- Not sure

10. Echocardiography is the most **accurate** method used at the bedside to establish or exclude a diagnosis of *pulmonary hypertension* in infants with BPD.

- Agree
- Disagree
- Not sure

11. Heart catheterization should routinely be performed to confirm a diagnosis of *pulmonary hypertension* in infants with BPD.

- Agree
- Disagree
- Not sure

12. Echocardiography is the most **feasible** method used at the bedside to identify and serially monitor *right ventricular failure* in infants with BPD.

- Agree
- Disagree
- Not sure

13. Echocardiography is the most **accurate** method used at the bedside to establish or exclude a diagnosis of *right ventricular failure* in infants with BPD.

- Agree
- Disagree
- Not sure

14. Several comprehensive reviews of echocardiographic methods for identifying and monitoring *pulmonary hypertension* in infants with BPD have been published. However, a white paper or position paper is needed to describe the most feasible, consistently repeatable, and accurate methods for identifying and monitoring pulmonary hypertension in infants with BPD

- Agree
- Disagree
- Not sure

15. Several comprehensive reviews of the echocardiographic methods for identifying and monitoring *right ventricular failure* in infants with BPD have been published. However, a white paper or position paper is needed to describe the most feasible, consistently repeatable, and accurate methods for identifying and monitoring right ventricular failure in infants with BPD.

- Agree
- Disagree
- Not sure

16. A white paper or position paper should include an explanation of the strengths and limitations of each echocardiographic measurement of pulmonary hypertension and right ventricular failure in infants with BPD.

- Agree
- Disagree
- Not sure

17. A white paper or position paper should describe how well echocardiography can distinguish between different degrees of severity in *pulmonary hypertension* (mild, moderate, severe).

- Agree
- Disagree
- Not sure

18. A white paper or position paper should describe how well echocardiography can distinguish between different degrees of severity in *right ventricular failure* (mild, moderate, or severe).

- Agree
- Disagree

- Not sure

19. If shared with all stakeholders, a white paper or position paper might improve communication between those performing and interpreting echocardiograms, and those providing care for infants with BPD.

- Agree
- Disagree
- Not sure

Block 3

FOCUS 3: Ability of Echocardiography to Accurately Evaluate Pulmonary Hypertension and Right Ventricular Failure in Infants with BPD and No Cardiovascular Shunt Other than a Patent Foramen Ovale or Small Atrial Septal Defect

20. In infants with BPD, which of the following parameters can be measured serially with **reasonable ease** and **accuracy**, allowing them to be useful in the identification and monitoring of *pulmonary hypertension*? Check all that apply.

- [ ] Gradient of tricuspid valve regurgitation
- [ ] Peak gradient of pulmonary valve insufficiency
- [ ] End-diastolic gradient of pulmonary valve insufficiency
- [ ] A subjective assessment of septal flattening
- [ ] Pulmonary artery acceleration time
- [ ] Pulmonary artery acceleration time, indexed for heart rate or ejection time
- [ ] End-systolic left ventricular eccentricity index
- [ ] Maximum left ventricular eccentricity index
- [ ] Right ventricular anterior or inferior wall thickness
- [ ] None of the above
- [ ] Not sure
- [ ] Other

21. In infants with BPD, which of the following parameters can be measured serially with **reasonable ease** and **accuracy**, allowing them to be useful in the identification and monitoring of *right ventricular failure*? Check all that apply.

- ☐ Tricuspid Annular Plane Systolic Displacement (TAPSE)
- ☐ Right Ventricular Fractional Area Change (FAC)
- ☐ Tissue Doppler imaging
- ☐ Right Ventricular Strain
- ☐ BNP or NT-proBNP
- ☐ None of the above
- ☐ Not sure
- ☐ Other

22. Echocardiography can accurately estimate the *systolic pulmonary arterial pressure* well enough to clearly distinguish between a normal, mildly increased, moderately increased, or severely increased pressure.

- ○ Agree
- ○ Disagree
- ○ Not sure

23. Echocardiography may not be able to accurately estimate the systolic pulmonary arterial pressure well enough to distinguish between a normal, mildly increased, moderately increased, or severely increased pressure.
However, it can be used to distinguish whether an infant with BPD has a *normal or a greater than normal systolic pulmonary arterial pressure.*

- ○ Agree
- ○ Disagree
- ○ Not sure

24. Echocardiography can accurately describe right ventricular function well enough to clearly distinguish between *normal, mildly decreased, moderately decreased, or severely decreased right ventricular systolic function*.

- ○ Agree
- ○ Disagree

- Not sure

25. Echocardiography may not be able to accurately estimate right ventricular function well enough to distinguish between normal, mildly decreased, moderately decreased, or severely decreased right ventricular function.
    However, it can be used to distinguish whether an infant with BPD has *normal or less than normal right ventricular systolic function.*

- Agree
- Disagree
- Not sure

26. What estimated systolic pulmonary arterial pressure by echocardiography should be used as a cutoff to raise concern for the presence of *pulmonary hypertension* in an infant with BPD? Check one response.

- 25 mmHg
- 26-30 mmHg
- 31-35 mmHg
- 36-40 mmHg
- > 40 mmHg
- Other

27. What range of estimated systolic pulmonary arterial pressure by echocardiography is consistent with **mild** pulmonary hypertension in an infant with BPD? Check all that apply.

- 25 - 30 mmHg
- 31 - 35 mmHg
- 36 - 40 mmHg
- 41 - 45 mmHg
- > 45 mmHg
- Other

28. What range of estimated systolic pulmonary arterial pressure by echocardiography is consistent

with **moderate** pulmonary hypertension in an infant with BPD? Check all that apply.

- [ ] 36 - 40 mmHg
- [ ] 41 - 45 mmHg
- [ ] 46 - 50 mmHg
- [ ] 51 - 55 mmHg
- [ ] 56 - 60 mmHg
- [ ] > 60 mmHg

29. What range of estimated systolic pulmonary arterial pressure by echocardiography is consistent with **severe** pulmonary hypertension in an infant with BPD? Check all that apply.

- [ ] 46 - 50 mmHg
- [ ] 51 - 55 mmHg
- [ ] 56 - 60 mmHg
- [ ] 61 - 65 mmHg
- [ ] > 65 mmHg
- [ ] Equal to the systolic systemic arterial pressure
- [ ] Greater than the systolic systemic arterial pressure
- [ ] Any pressure associated with right ventricular failure
- [ ] Other

30. Echocardiography can be used to determine whether the patient has a systolic pulmonary pressure that is < 0.25, 0.25-0.50, 0.50-0.75, 0.75-1.00, or > than the systolic systemic blood pressure.

- ○ Agree
- ○ Disagree
- ○ Not sure

31. In addition to the ratio of systolic pulmonary to systolic systemic arterial pressure, you must factor in the systolic systemic blood pressure measurement during an echocardiogram to estimate the actual systolic pulmonary arterial pressure and diagnose pulmonary hypertension.

- ○ Agree
- ○ Disagree
- ○ Not sure

Block 4

FOCUS 4: Impact of Echocardiographic Measurements on the Care of Infants with BPD

32. Which echocardiographic estimates of the severity of *pulmonary hypertension* need to be reported in order to guide clinical decisions about the care of infants with BPD. Check all that apply.

- [ ] Systolic pulmonary arterial pressure
- [ ] Mean pulmonary arterial pressure
- [ ] Diastolic pulmonary arterial pressure
- [ ] Ratio of systolic pulmonary arterial pressure to systolic systemic arterial pressure
- [ ] Pulmonary vascular resistance
- [ ] No quantitative measurements are needed. A subjective assessment of no, mild, moderate or severe pulmonary hypertension is sufficient.
- [ ] Other

33. How high does a patient's estimate of *systolic pulmonary arterial pressure* need to be to influence care? For example, at what level would you consider starting a medication to treat pulmonary hypertension or adjust respiratory support? Check all that apply.

- [ ] 26-30 mmHg
- [ ] 31-35 mmHg
- [ ] 36-40 mmHg
- [ ] 41-45 mmHg
- [ ] 46-50 mmHg
- [ ] 51-55 mmHg
- [ ] 56-60 mmHg
- [ ] > 60 mmHg
- [ ] Any pressure associated with abnormal physical findings
- [ ] Any pressure associated with right ventricular failure
- [ ] Other

34. What are the system-related, patient-related, and echocardiography-related obstacles to obtaining the measurements for pulmonary hypertension or right ventricular failure in infants with BPD? Please respond in the text box.

35. Please add any other comments regarding echocardiography for infants with BPD you'd like to communicate in the text box below.

Office of Institutional Research and Decision Support
oir@emory.edu

Powered by Qualtrics
